# Comparison of Total Joint Replacement Rate Between Patients With Hemophilia A and Patients With Hemophilia B: A Population-Based and Retrospective Cohort Study

**DOI:** 10.1177/1076029618794294

**Published:** 2018-09-13

**Authors:** Wen-Ya Lin, Jiaan-Der Wang, Yu-Tse Tsan, Wei-Cheng Chan, Kwok-Man Tong, Shin-Tsu Chang, Yuan-Yang Cheng

**Affiliations:** 1 Department of Pediatrics, Taichung Veterans General Hospital, Taichung, Taiwan; 2 Center for Rare Disease and Hemophilia, Taichung Veterans General Hospital, Taichung, Taiwan; 3 School of Medicine, China Medical University, Taichung, Taiwan; 4 Division of Occupational Medicine, Department of Emergency Medicine, Taichung Veterans General Hospital, Taichung, Taiwan; 5 Institute of Occupational Medicine and Industrial Hygiene, College of Public Health, National Taiwan University, Taipei, Taiwan; 6 School of Medicine, Chung Shan Medical University, Taichung, Taiwan; 7 Department of Orthopedics, Taichung Veterans General Hospital, Taichung, Taiwan; 8 Department of Physical Medicine and Rehabilitation, Tri-Service General Hospital, School of Medicine, National Defense Medical Center, Taipei, Taiwan; 9 Department of Physical Medicine and Rehabilitation, Taichung Veterans General Hospital, Taichung, Taiwan

**Keywords:** hemophilia A, hemophilia B, incidence, total joint replacement

## Abstract

Recurrent hemarthrosis in patients with hemophilia (PWH) results in chronic arthropathy requiring total joint replacement (TJR). This study aimed to compare the difference in TJR rate between patients with hemophilia A (HA) and hemophilia B (HB). A final total of 935 PWH (782 HA and 153 HB) without inhibitors were collected from the Taiwan’s National Health Insurance Research Database between 1997 and 2013. Demographics, clinical characteristics, and TJR rate were compared between the 2 groups. The annual use of clotting factor concentrate was not different between HA and HB groups (*P* = .116). The rate of comorbidities except for 29 PWH having HIV who were all in the HA group was also not different between the 2 groups. A total of 99 (10.6%) PWH had undergone 142 TJR procedures during the study period. All of them had received on-demand therapy. No difference was found in the cumulative incidence of TJR between HA and HB (*P* = .787). After adjusting for various confounders including age, pyogenic arthritis, and HIV infection, no increased risk of TJR was found in patients with HA versus Patients with HB (hazard ratio: 0.92, 95% confidence interval 0.54-1.58). This finding suggests that the rate of TJR between patients with HA and HB is not significantly different.

## Introduction

Inherited hemophilia A (HA) and hemophilia B (HB) are X-linked bleeding disorders characterized by a deficiency or absence of clotting factor VIII (HA) or IX (HB). Residual level of clotting factors in plasma defines the degree of hemophilia: <1% normal severe, 1% to 5% moderate, or 5% to <40% mild.^1^ Incidence of hemophilia is estimated to be 1 in 5000 male live births in HA and 1 in 30 000 in HB.^2,3^ With medical advances, life expectancy of hemophilia has improved markedly. Morbidities include bleeding into soft tissues, joints, and other organs with sequelae have been increasingly identified.^4^ Joint bleeding is the hallmark of severe hemophilia,^5^ and recurrent episodes result in hemophilic arthropathy characterized by synovial hypertrophy and cartilage damage, leading to progressive joint damage and irreversible deformities.^6,7^ Total joint replacement (TJR) has been performed in case of severe diseases with failed conservative measures to alleviate pain and restore joint functional deficits in PWH.^8^ Therefore, the rate of TJR in HA and HB may reflect clinical disease severity. Most studies have reported increased joint bleeding in patients with HA, with increased number of surgical procedures to correct musculoskeletal complications.^9–13^ In a retrospective cohort study and systemic literature review, Tagariello et al reported an increased risk of joint arthroplasties in patients with HA.^9^ However, there are fewer studies favoring a similar risk of joint bleeds or arthroplasty in HA and HB.^14,15^ Thus, it is not yet conclusive whether HB is clinically less severe than HA.^16–19^ This large-scale, population-based study aimed to compare the cumulative incidence of TJR between patients with HA and HB.

## Methods

### Data Sources

This study was approved by the Institutional Review Board of Taichung Veterans General Hospital in Taiwan. Analysis was performed on data released from the Taiwanese National Health Insurance Research Database (NHIRD). The National Health Insurance (NHI) Program was implemented in Taiwan on March 1, 1995. This program provides compulsory and universal health insurance coverage for nearly 100% of the population of around 23.5 million Taiwanese. The NHIRD provides a wide range of information, including ambulatory and hospitalization care files as well as registration records for research purpose. Each patient file in the NHIRD includes an encrypted personal identification number, sex, date of birth, date of enrollment, and medical claims made. The information on medical claims consists of prescription use, including drug name, dosage, and total expenditure, orders, diagnoses, and dates.

### Identification of Study Cohorts

**Figure 1.**
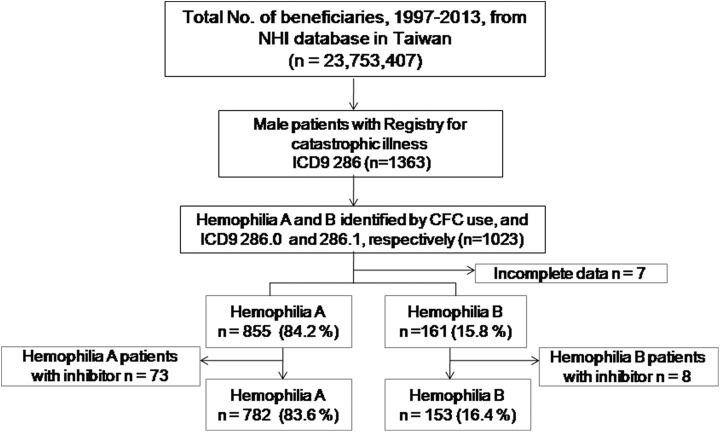
Patient selection. CFC indicates clotting factor concentrate; ICD-9, International Classification of Diseases, ninth revision; NHI, National Health Insurance.

In this study, data on patients with HA and HB groups were obtained from the Registry of Catastrophic Illness Database, a subdivision of the NHIRD. In this database, each newly diagnosed and registered case of hemophilia must be certified by 2 clinicians, and such individuals are eligible to receive free treatment with clotting factor concentrates (CFCs) according to the NHI guideline. The diagnostic code in this database is based on the system used by *the International Classification of Diseases, Ninth Revision, Clinical Modifications* (ICD-9-CM). We extracted data using *ICD-9* 286.0 for patients with HA and 286.1 for patients with HB from January 1, 1997, through December 31, 2013, to conduct the study. This time period was chosen for comparability, since all adult patients during this period had been treated under on-demand CFC replacement regimen, of which total reimbursement became available in 1997, whereas total reimbursement for prophylaxis of adult patients was initiated later in July 2014. PWH who had been treated with bypass agents were considered as having inhibitors to CFCs and were excluded from the study. Figure 1 demonstrates a final recruitment of 935 PWH in this analysis.

### Classification

The accuracy of diagnosis of major diseases in the NHIRD has been validated.^20,21^ But, an important limitation is that patients’ clotting factor levels are not recorded in the databases. Since NHIRD does not archive laboratory results such as plasma level of coagulation factor, classification was determined by the frequency of replacement therapy.^22^ PWH were thus subdivided into 3 groups according to the mean of the frequency of replacement therapy received. These included PWH requiring replacement therapy more than 2 times per year, PWH requiring replacement therapy <2 times per year, and the remaining group was PWH who did not require CFCs during daily life or only required CFCs treatment during the perioperative period.

### Outcome Measures

The primary objective of this retrospective cohort study was to compare TJR rate between patients with HA and patients with HB. We used order codes to identify PWH who underwent TJR: These included total hip replacement (THR; order code 64162B), total knee replacement (TKR; order code 64164B), total shoulder replacement (order code 64163B), total elbow replacement (order code 64165B), and total ankle replacement (order code 64167B). Clinical data and radiological images of PWH scheduled to undergo TJR must be evaluated by physicians of NHI bureau before the surgery. After approval, medical cost of TJR during perioperative period can be reimbursed. These order codes are used to apply for the surgical fee by hospitals. Therefore, indications of TJR are standardized, and the extracted data are well validated.

### Comorbidities

We identified PWH with comorbidities using *ICD-9* codes in a single inpatient setting or 3 or more outpatient visits; these included viral infection with HIV infection (ICD-9 code 42), hepatitis B virus (HBV) infection (*ICD-9* codes 0702-0704), and hepatitis C virus (HCV) infection (*ICD-9* codes 0707-0709, 07041-07042, 07044-07045, 07051-07052, and 07054-07055), pyogenic arthritis (*ICD-9* codes 711.0, 711.4, 711.6, and 711.9), hypertension (*ICD-9* code 401), ischemic heart disease (*ICD-9* codes 410-414), ischemic stroke (*ICD-9* codes 401–405), diabetes mellitus (*ICD-9* codes 250), and hyperlipidemia (*ICD-9* code 272).

### Statistical Analysis

All statistical analyses were performed using SAS software (version 9.2; SAS Institute Inc, Cary, North Carolina), and the significance level was set at .05. Descriptive data are presented as means and standard deviations. The frequencies were calculated by direct counting. The differences in demographics, clinical characteristics, and comorbidities between the 2 groups were analyzed using the *χ*^2^ test for categorical variables and the *t* test for continuous variables. Kaplan-Meier method was used to plot the cumulative incidence of TJR, and the log-rank test was used to examine the difference between the 2 groups. The rate of TJR in the 2 groups was compared by means of Cox regression models; this was the primary analysis with the hazard ratio as the primary effect measure. Potential confounding variables examined for their association with TJR defined as a priori were age, HIV infection, and pyogenic arthritis.

### Sensitivity Analysis

To examine potential effect modifiers, we conduct analyses stratified by groups with and without replacement therapy, HBV infection, HCV infection, diabetes mellitus, hypertension, and ischemic heart disease. These sensitivity analyses were applied to assess the difference and consistency between the hemophilia type and the risk of TJR.

## Results

A total number of 1023 male PWH were identified during this study period, and 7 were excluded due to incomplete data. PWH were subdivided into 855 HA (84.2%) and 161 HB (15.8%). After excluding 81 PWH with inhibitors to clotting factors, a final total of 935 PWH (782 patients with HA and 153 patients with HB) were enrolled (Figure 1).

**Figure 2.**
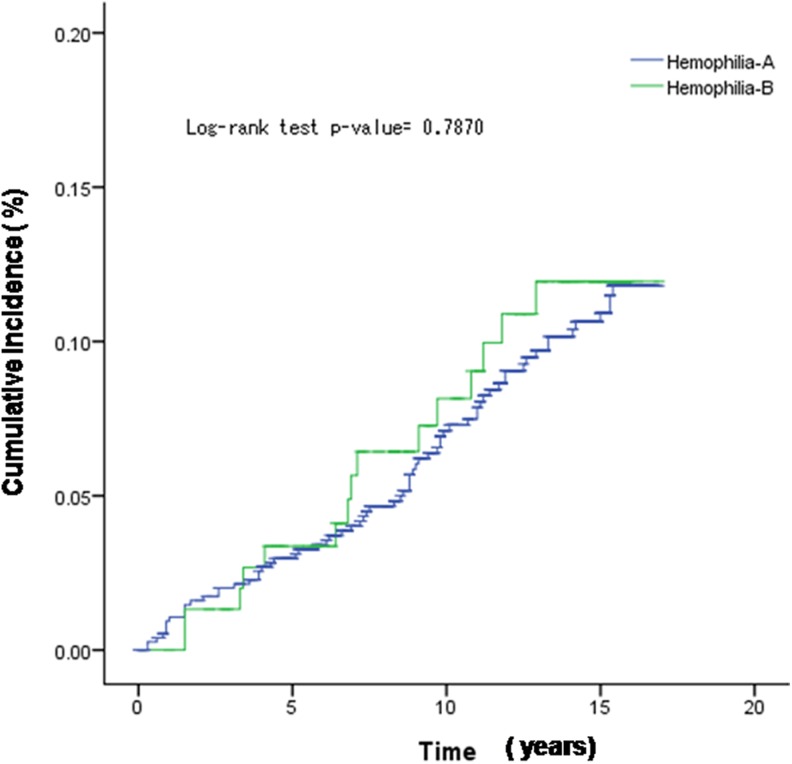
The Kaplan-Meier estimated cumulative incidence of total joint replacement in patients with hemophila A and hemophilia B.

Table 1 shows demographics, clinical characteristics, and TJR rate of enrolled patients with HA and HB. The age at end of study for HA group and HB group was 35.1 and 31.6 years, respectively (*P* = .023). A total of 101 PWH did not need replacement therapy during daily life, and these included 11.4% of patients with HA and 7.8% of patients with HB. In addition, 84.0% of patients with HA and 89.5% of patients with HB required replacement therapy more than 2 times per year. A total of 29 PWH had coexisting HIV infection, and all were in the HA group. Comparison of other comorbidities were not significantly different between HA and HB. We identified that 99 (10.6%) of 935 PWH required TJR, and no statistical difference in rate of TJR found between patients with HA (10.6%) and patients with HB (10.5%) between 1997 and 2013 (*P* = .954). Importantly, the cumulative incidence of first TJR between patients with HA and patients with HB by the unadjusted Kaplan-Meier analysis showed no significant difference in log-rank test (*P* = .787; Figure 2).

**Table 1.** Demographic, Clinical Characteristics, and Rate of Total Joint Replacement Between Hemophilia A and Hemophilia B.

	Total (n = 935), n (%)	Hemophilia A (n = 782), n (%)	Hemophilia B (n = 153), n (%)	*P* value
Age at study end, years, mean (SD)	34.6 (17.3)	35.1 ± 17.5	31.7 ± 16.4	.023^a^
Follow-up time/person, years	10 718.8	8901.2	1817.6	
Mean (SD)	11.5 ( 4.9)	11.4 (4.9)	11.9 (4.5)	.929
Frequency of replacement therapy				.108
Not required	101 (10.8)	89 (11.4)	12 (7.8)	
Less than 2 times/year	40 (4.3)	36 (4.6)	4 (2.6)	
More than 2 times/year	794 (84.9)	657 (84.0)	137 (89.5)	
Annual CFC use/person (IU), mean (SD)	90 567 (396 758)	96 630 (433 902)	61 241 (86 912)	.116
Comorbidity				
Hepatitis B virus infection	71 (7.6)	59 (7.5)	12 (7.8)	.899
Hepatitis C virus infection	226 (24.2)	191 (24.4)	35 (22.9)	.682
HIV infection	29 (3.1)	29 (3.7)	0 (0)	.016^a^
Hypertension	153 (16.4)	127 (16.2)	26 (17.0)	.818
Ischemic heart disease	43 (4.6)	32 (4.1)	11 (7.2)	.094
Ischemic stroke	46 (4.9)	37 (4.7)	9 (5.9)	.547
Hyperlipidemia	79 (8.5)	64 (8.2)	15 (9.8)	.510
Diabetics mellitus	59 (6.3)	51 (6.5)	8 (5.2)	.548
Pyogenic arthritis	45 (4.8)	39 (5.0)	6 (3.9)	.573
Mortality	114 (12.2)	98 (12.5)	16 (10.5)	.473
Total joint replacement	99 (10.6)	83 (10.6)	16 (10.5)	.954

Abbreviations: CFC, clotting factor concentrate; HIV, human immunodeficiency virus.

The frequencies were calculated by direct counting. The differences in demographics, clinical characteristics, and comorbidities between the 2 groups were analyzed using the χ^2^ test for categorical variables and the t test for continuous variables. Note: ^a^*P* value < 0.05.

Clinical characteristics of PWH requiring TJR were further analyzed in Table 2. The mean age of PWH undergoing first TJR was 37.2 years. Among these patients with TJR, 80 (96.4%) of 83 patients with HA and 15 (93.8%) of 16 patients with HB who had TJR were treated with CFC more than 2 times per year. Four individuals who had no frequency of using CFC before TJR did not have severe hemophilia, and the cause of TJR was degenerative joint diseases. The amount of annual CFC use was not different between the 2 groups. All 15 PWH with pyogenic arthritis were found in the HA group.

**Table 2.** Demographic and Clinical Characteristics of Patients with Hemophilia A and B with Total Joint Replacement.

	Total (n = 99)	Hemophilia A (n = 83)	Hemophilia B (n = 16)	*P* value
	n	n (%)	n (%)	
Age at first TJR, years, mean (SD)	37.2 (12.9)	37.1 (11.8)	37.4 (16.4)	.929
Frequency of replacement therapy				.625
Not required	4	3 (3.6)	1 (6.3)	
Less than 2 times/per year	0	0 (0)	0 (0)	
More than 2 times/per year	95	80 (96.4)	15 (93.8)	
Annual CFC use/person (IU), mean ± SD	597 537 (1 417 48)4	670 167 (1 568 592)	286 269 (154 355)	.198
Comorbidity				
Hepatitis B virus infection	11	10 (12.1)	1 (6.3)	.499
Hepatitis C virus infection	59	52 (62.7)	7 (43.8)	.158
HIV infection	3	3 (3.6)	0 (0)	.440
Pyogenic arthritis	15	15 (18.1)	0 (0)	.064
Site of first TJR				
Total hip replacement	20	17 (20.5)	3 (18.8)	.874
Total shoulder replacement	1	0 (0)	1 (6.3)	.022^a^
Total knee replacement	71	60 (72.3)	11 (68.8)	.773
Total elbow replacement	2	2 (2.4)	0 (0)	.530
Total ankle replacement	0	0 (0)	0 (0)	–
Other joint replacement	5	4 (4.8)	1 (6.3)	.811
Total number of TJR	142	116	26	
Mean number of TJR, mean (SD)	1.4 (0.7)	1.4 (0.6)	1.6 (1.0)	.400
Age at study end, mean (SD)	45.4 (12.0)	45.5 (11.2)	44.6 (15.9)	.818
Mortality	6	5 (6.0)	1 (6.3)	.972

Abbreviations: CFC, clotting factor concentrate; HIV, human immunodeficiency virus; TJR, total joint replacement.

The frequencies were calculated by direct counting. The differences between the 2 groups were analyzed using the *χ*^2^ test for categorical variables and the t test for continuous variables. Note: ^a^*P* value < 0.05.

During the study period, the total number of TJR was 142, with 116 procedures performed in HA group. The mean number of TJR was 1.40 per patient with HA and 1.62 per patient with HB . In both groups, TKR was the most common operation of first TJR, accounting for 72.3% and 68.8% of patients with HA and HB, respectively. The second most common of first TJR was THR, found in 20.5% and 18.8% of HA and HB groups. A total of 6 (5 of HA and 1 of HB) of these 99 PWH died during the study period.

**Figure 3.**
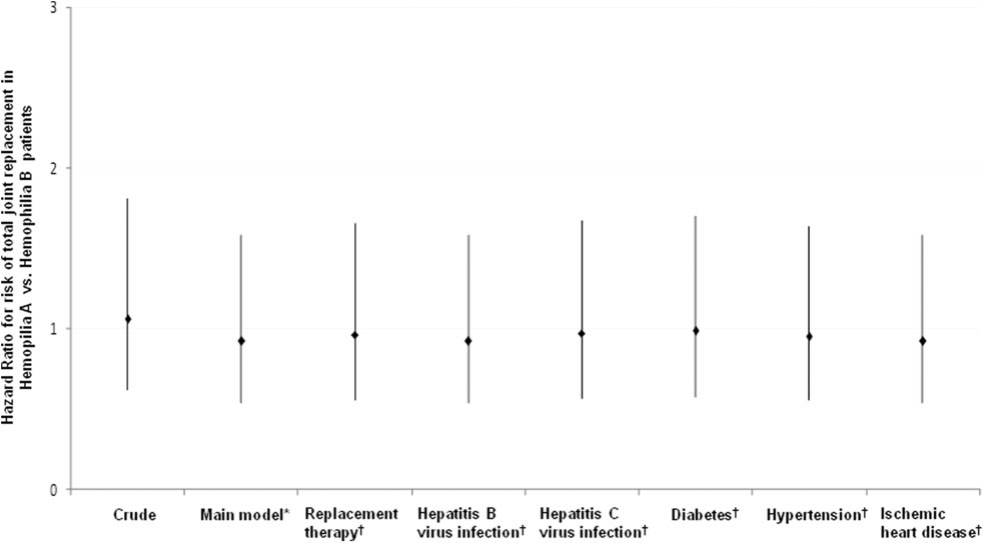
Sensitivity analyses of total joint replacement rate between patients with hemophilia A and hemophilia B. *Main model is adjusted for age, human immunodeficiency virus infection, and pyogenic arthritis. **^†^**The models are adjusted for covariates in the main model as well as each additional listed covariate.

After adjusting for various confounders, including age, HIV infection, and pyogenic arthritis, the risk of TJR in HA versus HB group was not significantly higher (hazard ratio, 0.92, 95% CI 0.53–1.58, *P* = .756; Figure 3). To assess whether the overall results could have been influenced by replacement therapy, hepatitis virus infection, diabetes, hypertension, or ischemic heart disease, the sensitivity analysis performed also showed no deviations from the overall estimate.

## Discussion

The result of this retrospective cohort study provides evidence that the risk of TJR is not different between patients with HA and HB. Consistent with our findings are the result of the study conducted by den Uijl and colleagues. This longitudinal data concerning 5094 treatment-years for 252 patients with severe HA and 30 patients with severe HB having similar parameters of bleeding pattern and clotting factor use showed no significant difference in onset and frequency of joint bleeding or rate of arthroplasties.^14^ A similar hemophilia severity score (bleeding score, joint score, and use of CFC) was also reported by Tagliaferri et al in Italian patients with HA and HB.^23^ In addition, van Dijk et al reported age of first joint bleed is inversely related to arthropathy and treatment required and thus could pose as an indicator of clinical severity in PWH^24^; this was found to be similar between 582 children with severe HA and 76 children with severe HB.^15^

In contrast, there are some conflicting results on TJR rate between patients with HA and HB. Tagariello et al performed a retrospective analysis of data collected from 29 Italian hemophilia centers, it was found that 1770 patients with severe HA had a >3-fold higher risk of undergoing joint arthroplasties in comparison to 319 patients with severe HB. After adjustment for HIV infection, HCV infection, and inhibitor status in a Cox regression model, the results were not affected.^9^ A higher percentage of patients with HA underwent surgery for correction of musculoskeletal complication than HB patients was found in a 3-year study conducted by Nagel et al (14.7%; 10 of 68 patients with HA vs 4.7%; 1 of 21 patients with HB).^11^ In the Universal Data Collection database, the prevalence of hip abnormalities in patients with HA was also higher than that in patients with HB (18%; 1125 of 6419 vs 14%; 247 of 1773, *P* = .0003), although the differences in loss of range of motion were not significant across all the severity groups of PWH.^25^

The hallmark of hemophilia is joint bleeding (encompass 90% of all bleeding episodes); common sites involved are ankles, knees, and elbows.^5,26,27^ Recurrent bleeding contributes to chronic arthropathy. Surgical procedures with TJR are performed in PWH with severe joint damage and failure of conservative therapy. However, it is difficult to use TJR as the sole variable to describe the difference in severity between the 2 groups. As hemophiliac arthroplasty pose as treatment for an end-stage event, there are multiple variables that could influence progression to this end-stage arthropathy and thus bias results. These include patients’ genetic background, biological half-life and in vivo recovery of CFC, inhibitor status, bleeding frequency, use of prophylaxis or on-demand therapy, age of onset at first joint bleed, and comorbidities. In addition, the criteria for TJR may vary within different clinical departments.

Patients with hemophilia having inhibitors to clotting factors have wide variety of clinical severity and totally different bleeding phenotype from PWH with no inhibitors. The presence of inhibitors often discourages physicians from performing TJR. Therefore, in order to prevent the introduction of this selection bias, all PWH with presence or past history of inhibitors were excluded from this study. Several comorbid diseases, such as HCV and HIV infection in PWH, aggravate clinical disease severity; this may influence decision for or timing of surgical intervention and contribute to mortality,^4,28^ thus ultimately biasing number of PWH receiving joint arthroplasties. Furthermore, local diseases of the joint such as pyogenic arthritis can also accelerate hemophilic arthropathy and influence surgical outcome.^20,29^ Thus, these confounders were identified, and after adjustments by Cox regression analysis, no significant increased risk of TJR in patients with HA versus Patients with HB were found in this model.

Prophylactic rate in PWH may reflect difference of severity in the bleeding phenotype. Lower prophylactic rate at each severity in patients with HB compared to patients with HA was reported according to a survey of 2663 Canadian children and adults with hemophilia.^30^ On the other hand, PWH with prophylaxis changes the severity of hemophilia, affects the progression of arthropathy, and the need for surgical intervention. In this cohort study, all PWH with TJR received on-demand clotting factor therapy, cost of which was reimbursed by Taiwanese NHI. Prophylactic CFC treatment was provided and reimbursed by the Taiwanese NHI only from 2014. Therefore, TJR rate in this study is not biased by prophylactic CFC use.

We performed further analysis on the 99 PWH with TJR to investigate whether the 2 groups differ in certain clinical characteristics, which could alter the decision for or timing of TJR treatment. It was found that the majority of both of these patients with HA (96.4%) and patients with HB (93.8%) required replacement therapy more than 2 times per year. This finding implied that PWH with TJR had severe type of hemophilia and required more replacement therapy. The age at first TJR was not different between HB patients with HA and, with a similar mean of 1.63 TJR procedures during the 16-year of follow-up. Although spontaneous bleeding into hip joint is less common than knee, secondary degenerative changes often develop and contribute to joint damage in PWH.^31,32^ TKR is well documented to be the treatment of choice for patients with severe knee arthropathy, and there has also been increasing report of successful pain relief and functional improvement after THR.^32,33^ Consistent with these findings, most common sites for TJR were the knee, thereafter followed by hip in both the studied HA and HB groups. Despite elbow and ankle being common sites of bleeding, less number of PWH with elbow joint replacement (2 patients with HA), and no ankle joint replacement were found in our study. Probable explanations include that severe elbow arthropathy can be tolerated for long time, and eventual bone chronic transformation is so great that effective prosthesis placement may be difficult.^26^ With regard to the ankle, total ankle replacement pose as uncertain treatment option with insufficient follow-up, with other surgical procedures including arthrodesis being more commonly recommended and practiced.^26^ As ankle artificial prosthesis also cost much, and is not reimbursed by Taiwanese NHI, this could also have some influence.

In summary, there was no difference in clinical characteristics between patients with HA and HB undergoing TJR.

## Limitations

A key advantage of this study is that data are extracted from population-based analysis and is thus highly representative of the general population. However, there are certain limitations that need to be considered. First, PWH were enrolled from the catastrophic illness database of the NHIRD. Mild hemophilia was not captured well in this study. This could have resulted in selection bias of PWH with more severe clinical disease. Second, detailed clinical information such as patients’ baseline clotting level were unavailable from the NHIRD; thus, we extracted use of CFCs in PWH, as patient classification. Third, chronic arthropathy is associated with recurrent hemarthrosis, but the number of annual joint bleeding is not reflected in this database. In addition, the duration of the particular treatment that could reflect severity of arthropathy is not retrievable. Finally, lack of genetic data and biological half-life and in vivo recovery of CFC could result in a bias of comparison between the 2 groups. However, as TJR pose as treatment for hemophilic arthropathy, this study has focused on comparing incidence of TJR between HA and HB groups after adjusting multiple variables.

## Conclusions

The rate of TJR between patients with HA and HB is not significantly different. Even after adjusting for confounders, no increased risk of TJR is found in the HA group. TJR is performed in hemophilic arthropathy, usually after failure of conservative therapy; thus, this may reflect clinical disease severity in PWH.
